# Antioxidant and Anticancer Assessment and Phytochemical Investigation of Three Varieties of Date Fruits

**DOI:** 10.3390/metabo13070816

**Published:** 2023-07-03

**Authors:** Ahmed S. Abdelbaky, Mohamed A. Tammam, Mohamed Yassin Ali, Marwa Sharaky, Khaled Selim, Wael M. Semida, Taia A. Abd El-Mageed, Mohamed Fawzy Ramadan, Hesham F. Oraby, Yasser M. Diab

**Affiliations:** 1 Department of Biochemistry, Faculty of Agriculture, Fayoum University, Fayoum 63514, Egypt; asm03@fayoum.edu.eg (A.S.A.); mya02@fayoum.edu.eg (M.Y.A.); ymd00@fayoum.edu.eg (Y.M.D.); 2 Pharmacology Unit, Cancer Biology Department, National Cancer Institute, Cairo University, Giza 11796, Egypt; marwa.sharaky@nci.cu.edu.eg; 3 Department Food Science and Technology, Faculty of Agriculture, Fayoum University, Fayoum 63514, Egypt; kas00@fayoum.edu.eg; 4 Department of Horticulture, Faculty of Agriculture, Fayoum University, Fayoum 63514, Egypt; wms00@fayoum.edu.eg; 5 Department of Soil and Water, Faculty of Agriculture, Fayoum University, Fayoum 63514, Egypt; taa00@fayoum.edu.eg; 6 Department of Clinical Nutrition, Faculty of Applied Medical Sciences, Umm Al-Qura University, Makkah 21955, Saudi Arabia; 7 Deanship of Scientific Research, Umm Al-Qura University, Makkah 21955, Saudi Arabia

**Keywords:** *Phoenix dactylifera* L., antioxidants, cancer cell lines, HPLC-DAD, phenolics, ethyl acetate extract

## Abstract

Date palm (*Phoenix dactylifera* L.) fruits contain high concentrations of phenolic compounds, particularly flavonoids and other micronutrients, which impact human health due to their potent antioxidant, anti-inflammatory, and anticancer characteristics. In the present study, the effect of ethyl acetate, hydroethanol, hydromethanol, and aqueous extract from three date palm varieties (i.e., Ajwa, Siwi, and Sukkari) on phytochemical profiles and antioxidant and anticancer activities was investigated. Fruit extracts were screened for their antioxidant activity using the DPPH· method. Phenolic constituents were quantified and identified using HPLC-DAD. Extracts (ethyl acetate, hydroethanol, and hydromethanol) were assessed for cytotoxicity on nine human cancer cell lines, i.e., MG-63, HCT116, MCF7, MDA-MB-231, HEPG2, HUH7, A549, H460, and HFB4, using the sulphorhodamine-B (SRB) assay. Results showed that the ethyl acetate extract of the Sukkari fruits has the greatest antioxidant potential with an IC_50_ value of 132.4 ± 0.3 μg·mL^−1^, while the aqueous extract of Ajwa date fruits exhibited the lowest antioxidant effect with an IC_50_ value of 867.1 ± 0.3 μg·mL^−1^. The extracts exhibited potent to moderate anticancer activities against the investigated cancer cell line in a source-dependent manner. Methanol extract of Siwi fruits exhibited the most potent anticancer activity (IC_50_ = 99 ± 1.6 µg·mL^−1^), followed by the same extract of Sukkari fruits with an IC_50_ value of 119 ± 3.5 µg·mL^−1^ against the cell line of human breast cancer (MDA-MB-231). Additionally, principal component analysis (PCA) was investigated to determine the relationship among the investigated traits and treatments. Our findings reveal that date palm fruit-derived extracts are excellent sources of biologically active constituents and substantiate their potential use in new anticancer strategies from natural resources.

## 1. Introduction

Date palm is one of the main and most important commercial crops globally, with high economic benefits and excellent nutritional value in the Middle East, especially in the Arabian countries. Date fruit is distinguished by its high content of simple monosaccharides, e.g., fructose and glucose (65–85%) [1,2,3,4]. Recent studies have indicated that dates are a bio-supplier of dietary fiber and macro- and micro-elements [5,6]. Besides nutritional compounds, date fruits are considered prolific factories for bioactive natural compounds, including phenolic acids, flavonoids, tannins, terpenoids, carotenoids, and anthocyanins, which are well known to exhibit an extensive range of various biological activities such as *in vitro* antioxidants, antimicrobial, anti-inflammatory, anti-proliferative, and enzymatic activities [7,8,9]. In addition, the date palm has a long history of being used to address issues with the reproductive and endocrine systems [10].

Nowadays, antioxidants derived from natural sources are being used for a wide range of purposes because they are ecologically safe, culturally more acceptable, and less hazardous than synthetic ones [11,12]. Moreover, date fruits are considered efficient for their natural antioxidant effect due to their prominent level of polyphenolic compounds such as sinapic and ferulic acids, flavonoids, procyanidins, *p*-coumaric acid, and flavonoid glycosides, i.e., apigenin, quercetin, and luteolin [13,14].

These bioactive compounds must be extracted, identified, and quantified to develop functional foods and nutraceuticals. The extraction technique is an essential step in this process [15]. However, traditional extraction methods involve organic solvents, and cold or hot extraction has a variety of problems, including the inefficiency of the product’s quality, safety hazards, and environmental concerns [15]. Ultrasound-assisted extraction (UAE) is simple, rapid, and a better method since the antioxidant activity, phenolic, and flavonoid contents are higher [16].

According to the WHO, one of the main causes of death all over the world after cardiovascular diseases is cancer [17]. Among cancers, lung, breast, stomach, liver, and colorectal cancers are the leading causes of death yearly [17,18]. Natural compounds originating from plants are one of the main sources of potential anticancer drugs [19] due to their novel structures. Natural products exhibit extraordinary action against different forms of cancer [20]. Indeed, the discovery of new natural products with potential activity against various cancer cells showing no or non-significant toxicity for the normal cells becomes an urgent necessity because using organic materials to find new drugs is limited due to their toxicity or inactivity against the targeted cells.

Diet and lifestyle play a significant role in cancer prevention and treatment [21]. Date palm fruit can be considered an exceptional material for manufacturing healthy snack bars due to its richness in several nutritional compounds [22]. Carbohydrates with 40 to more than 80% represent the main component of dates. Dates’ carbohydrates consist mainly of reducing sugars existing in the form of monosaccharides, i.e., fructose, glucose, and mannose, as well as the disaccharide maltose and the non-reducing sugar sucrose, as well as a minute number of polysaccharides, mainly starch, cellulose, and *β*-glucans [23]. Furthermore, date fruits are a good source of high-quality dietary fibers, including cellulose, arabinoxylans, and *β*-glucans [1]. Moreover, with a 1.7 to 4.7% protein percentage, date fruits are considered a poor source of protein [24]. Furthermore, date fruits were found to contain high-quality amino acids, either essential, including lysine, histidine, isoleucine, and tryptophan, or non-essential, including proline and tyrosine [22]. Dates contain a very minute amount of lipids—almost less than 1% [25]. Additionally, date fruits are well known to be a good supplier of different vitamins, mainly vitamin B [22] and E in the form of tocopherols [23], vitamins C, K, and A in the form of lutein, and *β*-carotene, which is also found in date fruits but in relatively small amounts [26,27,28]. Date fruits contain a relatively high concentration of calcium, potassium, and magnesium, of which potassium is the most abundant [22]. Moreover, date fruits contain iron, copper, sodium, and phosphorus as well, but in very minute amounts [29]. It is worth mentioning that date fruits are considered to be a suitable product for those suffering from hypertension due to their high content of potassium and low concentration of sodium [30].

One of the extraordinary dates is the Ajwa date, which exists only in Madina, Saudi Arabia, with its high nutritional and medical value [8,13,14]. Ajwa dates have been labeled in folklore medicine to afford numerous health advantages, for instance, anticholesterolemic, antidiabetic, anti-inflammatory, antioxidant, hepato-protective, and anticancer properties [31,32].

The highest content of proteins and nutrition exists in the variety of Sukkari [33]. Furthermore, Sukkari dates contain the highest content of fat, ranging from 0.5–0.7% [28]. Additionally, Sukkari dates contain a high concentration of minerals when compared with other fruits such as mango and pomegranate [25]. It is considered a rich source of antimicrobial agents and antioxidant activity [34,35]. So far, only one report has dealt with the Siwi variety biologically and phytochemically [6].

Despite several studies on the date palm varieties as safe and edible natural resources, to date, there is no data regarding the phytochemical profiles of the diversity of chemical constituents and the antioxidant and anticancer activities of the effects of ethyl acetate extract of three varieties of date palm (Ajwa, Siwi, and Sukkari). So, the present study was conducted to assess the effects of ethyl acetate, hydroethanol, and hydromethanol, as well as aqueous fruit extracts of three date palm varieties (Ajwa, Siwi, and Sukkari), on phytochemical profiles, antioxidants, and anticancer activities, which are expected to substantiate their potential use in new anticancer strategies from safe and natural resources.

## 2. Materials and Methods

### 2.1. Chemicals and Reagents

All materials used for the experimental work, including extractions and the analytical protocols, have been purchased from various suppliers, i.e., gallic acid, rutin, DPPH·, Folin–Ciocalteu reagent, and aluminum chloride anhydrous were acquired from Sigma Aldrich (St. Louis, MO, USA). Ethyl acetate, methanol, and ethanol were from Advent Chembio Pvt. Ltd. (Mumbai, India) and have been distilled before use. Sodium carbonate was from Fluka (Buchs, Switzerland). Nineteen pure phenolic compounds (HPLC standards, 99.9%) for quantification were acquired from Sigma-Aldrich (Merck, Buchs, Switzerland).

### 2.2. Sample Collection

Ajwa and Sukkari date palm fruits were obtained from a date factory in Al-Madinah al-Munawwarah City, Saudi Arabia. Siwi date fruits were obtained from the date factory in Fayoum City, Egypt. The samples were randomly selected from a semi-dry batch without consideration for size, shape, color, or appearance. Seeds were removed from their fruits, air-dried at room temperature, then powdered by a laboratory blender (Bosch, 1600w, Grand Rapids, MI, USA), and stored in airtight bottles at −80 °C (refrigerator −80 °C, Labconco, Kansas City, KS, USA) until use.

### 2.3. Extract Preparation

**Figure 1:**
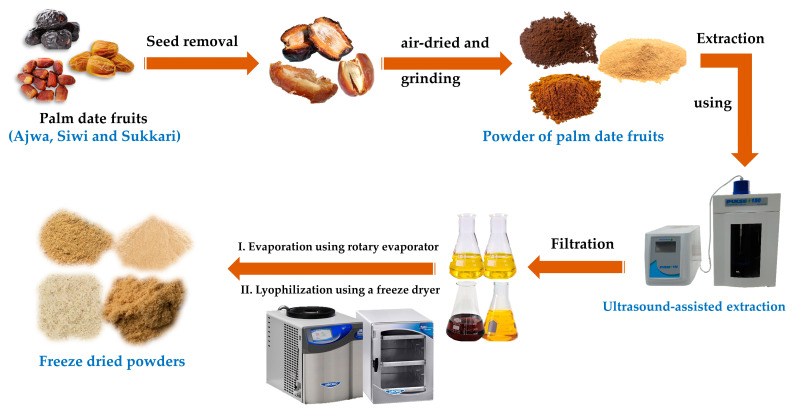
Extraction process of the three varieties of date samples.

A known amount of each sample was weighed (50 g/250 mL) and extracted individually with ethyl acetate (EtOAc), hydroethanol (EtOH/H_2_O, 70%), hydromethanol (MeOH/H_2_O, 80%), and water (H_2_O) in conical flasks. Using the ultrasound-assisted solvent extraction (UASE) technique, the extraction was carried out by placing the conical flasks in a Probe Sonicator homogenizer (Benchmark Scientific, Sayreville, NJ, USA, 150 W, 25 kHz) for 30 min [36]. The extracts were filtered off. The residue of each extract was extracted again, as mentioned above. Then the yields of both extractions were added together. Utilizing a rotary evaporator (BUCHI, R300, Flawil, Switzerland), they were evaporated under vacuum at 45 °C and then freeze-dried to dryness to obtain dried extracts, as shown in Figure 1. All extracts were used individually to evaluate their biological activities.

### 2.4. Chemical Composition

The chemical composition of the three date samples (moisture, ash, crude protein, lipid, and crud fiber content) was determined according to A.O.A.C. [36]. Moisture content was determined by transforming 10 g of crushed date bits into the dish that had already been oven dried. The sample was then placed in an oven at 110 °C until a constant weight was attained. The following formula was used to determine the moisture content percentage on a dry basis:

Moisture content (%) = Change in weight/weight of dry matter × 100.

The determination of proteins in terms of nitrogen was performed using the micro Kjeldahl method (Buchi, KjelFlex K-360, Flawil, Switzerland). The nitrogen value was converted to protein by multiplying by a factor of 6.25. The ash values were obtained by heating date fruit pulp (5 g) on the Bunsen burner until the formation of fumes became obvious, at which point the sample was moved into the furnace at 550 °C and remained in it until a gray-colored sample was formed [37]. After cooling the sample, the ash’s weight was determined. The ash content was determined using the formula below:

ASH (%) = weight of ash/weight of sample × 100.

The Anthrone method [38] was used to measure the total amount of sugar. A fresh date (0.5 g) was cooked in 10 mL of 80% ethanol before filtering. The amount of filtrate that was generated was 50 mL. Filtrates and anthrone reagent were added to a reaction mixture, which was then put into a water bath at 100 °C for 15 min. After 20 min of letting the reaction mixture lie at room temperature, the optimum density was measured at 620 nm. Similar procedures were used for the blank sample. The soluble sugars were calculated based on the standard curve. Crude fats were determined by the Soxhlet apparatus using n-hexane as a solvent.

### 2.5. Mineral Content Determination by ICP-OES

Powdered samples of the dates were subjected to acid digestion, and 0.5 g of powdered samples was digested with 3.5 mL each of conc. sulfuric acid and 30% H_2_O_2_ for 30 min after heating at 25 °C. Next, after cooling, 30% of the 1 mL of H_2_O_2_ aliquot was mixed and filtered. Using 20 mL of distilled water, the filtrate was diluted [39]. Then the mineral analyses were carried out using inductively coupled optical plasma emission spectrometry (ICP-OES) with synchronous vertical dual view (SVDV) to record the content of calcium (Ca), magnesium (Mg), phosphorous (P), potassium (K), iron (Fe), copper (Cu), zinc (Zn), manganese (Mn), and sodium (Na)) in three varieties of dates.

### 2.6. Determination of Amino Acids by HPLC-MS

The amino acid makeup of the analyzed date fruits was determined using the technique outlined by Jajic et al. [40]. One gram of each sample was dissolved and mixed well in 5 mL of distilled water (dH_2_O) and 5 mL of 6 M hydrochloric acid (HCl), then heated for 24 h at 100 °C, then filtrated, and the obtained filtrate was used for the analysis. HPLC analysis was conducted using an Agilent 1260 system (Agilent Technologies, Santa Clara, CA, USA), a 1260 binary pump equipped with a 1260 diode array fluorescence detector, and an Eclipse Plus C18 column (4.6 mm × 250 mm i.d., 5–378 μm), using (A) sodium phosphate dibasic and sodium borate buffer, pH 8.2 (A) and (B) ACN: MeOH: H_2_O 45:45:10, as the mobile phase. Using a gradient series of 98% A and 2% B (0 min); 98% A and 2% B (0.84 min); 43% A and 57% B (33.4 min); 0% A and 100% B 382 (33.5 min); 0% A and 100% B (39.3 min); 98% A and 2% B (39.4 min); 98% A and 2% B 383 (40.0 min) for the elution, column temperature was preserved at 40 °C. The DAD was observed at 338 nm (bandwidth 10 nm). The fluorescence detector was adjusted as follows: from 0 to 27 min at 340/450 nm (excitation/emission) and from 27 to 35 min at 266/306 (excitation/emission).

### 2.7. DPPH· Radical Scavenging Activity of Different Extracts of Dates

The DPPH· radical-scavenging activity (DPPH· RSA) of all the extracts was calculated by the 2,2-diphenyl-1-picrylhydrazyl (DPPH·) free radical [41]. The concentration that caused 50% inhibition of all the present free radical extracts (IC_50_) was calculated by the analysis of the linear regression of the dose-response curve, plotting inhibition percentage versus the concentration of the extract (10–320 µg·mL^−1^). All measurements were carried out in triplicate. Control experiments (a negative control assay using each solvent without dates palm extract and a positive control assay with L-ascorbic acid) were executed similarly and simultaneously as the dates palm extract assays.

### 2.8. Total Phenolic and Flavonoid Contents

The amount of total phenolic in the dates palm extracts was measured by the Folin–Ciocalteu reagent using the procedure of Yu et al. [42]. The total flavonoid content (TFC) in the date palm extracts was determined using the colorimetric technique [43].

HPLC analysis was performed for the identification of phenolic constituents by HPLC-DAD analysis on an Agilent 1100 series liquid chromatography pump equipped with an autosampler and a diode-array detector, using an Eclipse XDB-C18 (150 × 4.6 µm; 5 µm) with a C18 guard column (Phenomenex, Torrance, CA, USA), using acetonitrile (solvent A) and 2% acetic acid in water (*v*/*v*) (solvent B) as eluent, and a 0.8 mL/min flow rate for the whole run (70 min). The resulting peaks were detected simultaneously at 280 nm for the benzoic acid derivatives and 320 nm for the cinnamic acid derivatives. Using a 0.45 µm Acrodisc syringe filter (Gelman Laboratory, Ann Arbor, MI, USA), all samples were filtered before injection according to Kim et al. [44]. Peaks were recognized by comparing preservation times and UV data with standards.

### 2.9. Anticancer Activity of Different Extracts of Dates

Human osteosarcoma cell line (MG-63), human colon cancer cell line (HCT116), human breast cancer cell lines (MCF7 and MDA-MB-231), human hepatocellular cancer cell lines (HEPG2 and HuH7), human lung cancer cell lines (A549 and H460), and human normal melanocyte cell line (HFB4) were used for the screening and were obtained from the American Type Culture Collection (ATCC, Manassas, VA, USA) and were maintained at the Pharmacology Unit Cancer, Biology Department, National Cancer Institute (NCI), Cairo University, Cairo, Egypt, in DMEM containing 10% fetal bovine serum, 0.2% NaHCO_3_, and 1% penicillin-streptomycin. Every cell line was routinely incubated at 37 °C with 5% CO_2_ in a humid environment. The date palm extract’s cytotoxic effect on all tested cancer cell lines was evaluated using a sulphorhodamine-B (SRB) assay [45]. Fleetingly, cells were seeded at a density of 4 × 10^3^ cells/well in 96-wells. They were allowed to connect for 24 h before extract incubation, following the treatment of the cells with concentrations of 62.5, 125, 250, and 500 µg·mL^−1^ for MG-63, HCT116, MCF7, MDA-MB-231, HEPG2, HuH7, A549, H460, and HFB4 cells. A triplicate for each concentration was used, and incubation was extended for 48 h. Using DMSO as a control vehicle (1% *v*/*v*). After 48 h, cells were treated with 20% trichloroacetic acid to be fixed and stained with 0.4% SRB dye. Each well’s optical density (O.D.) was determined at 570 nm using an ELISA microplate reader (TECAN Sunrise™, Crailsheim, Germany). The mean survival fraction at each extract concentration was determined: O.D. of the treated cells divided by the control cells O.D.

### 2.10. Statistical Analysis

Using the statistical software program Genstat versions: 14.2 (VSN International Ltd., Oxford, UK), data were examined using the analysis of variance (ANOVA) approach. Utilizing the Duncan multiple range test (DMRT) with a significance level of 0.05, the variance between means was compared. The principal component analysis (PCA) was carried out utilizing the R version 4.2.3 statistical software package factoextra [46].

## 3. Results and Discussion

### 3.1. Date Fruit Chemical Composition

The average chemical composition of the three studied date varieties is presented in Table 1. Carbohydrates, protein, fat, fiber, and ash contents were shown on a dry weight basis. The moisture content of the studied varieties ranged between 17.31 ± 0.46 and 19.18 ± 0.50%, which is considered within the normal limits for semi-dry date varieties. Siwi had the highest moisture content, while Ajwa had the lowest, with no significant difference between Sukkari and the other two varieties. The findings are like those previously reported for dates from Saudi Arabia [47] and in agreement with those founded by Ramadan et al. and Selim et al. for Egyptian Siwi dates [48,49], but higher than those reported by Perveen and Bokahri for Sukkari dates [38].

Dates are well known for their high sugar content; the total sugar level of the samples ranged from 75.2 ± 1.75 to 80.2 ± 2.02%, with a significant difference between Ajwa and Siwi/Sukkari. The Ajwa date showed the lowest total carbohydrate content. The reducing sugars represented most of the sugars found in the studied date varieties, where the percentage of reducing sugars ranged between 71.5 ± 1.25 and 75.6 ± 1.05, and the Sukkari date recorded the highest percentage, while the lowest percentage was in the Ajwa variety. Our findings are similar to those described by Perveen and Bokahri [38] and greater than those indicated by Al-Tamim, Aldhafiri, and Siddeeg et al. for the Sukkari date [25,50,51]. The results are also consistent with those reported by Selim et al. [49] and higher than those reported by Ramadan et al. for the Siwi date variety [48]. Among the date fruit samples examined, the Ajwa date had a higher amount of ash, protein, and fiber than the other samples, while there was no significant difference between the Sukkari and Siwi date in their ash, protein, and fiber content. In addition, no significant variation was observed in lipids among all the tested samples. These findings are quite close to those described by [25,50,51] for Siwi, Ajwa, and Sukkari dates. The variances in the chemical composition of the investigated dates could be ascribed to differences in cultivar, soil fertility, growing environment, ripening stage, and harvest/postharvest practices.

### 3.2. Date Fruit Mineral Composition

Table 2 lists the average values for the tested date fruit samples’ macro- and micro-elements. The results proved that all the tested samples contained reasonable amounts of macro- and micro-elements, raising the dates’ nutritional value. Among different macro-elements, potassium was the predominant mineral element and ranged from (1038.1 ± 3.45–1118.6 ± 4.27 mg 100 g^−1^ DW) followed by phosphorus (109.9 ± 2.07–231.3 ± 6.25 mg 100 g^−1^ DW), magnesium (91.59 ± 3.52–151.1 ± 2.32 mg 100 g^−1^ DW), and calcium (55.73 ± 1.87–128.7 ± 3.22 mg 100 g^−1^ DW). The results support earlier research that showed dates had a high potassium concentration [52,53]. Regarding the microelements, iron concentration was the predominant micro-element in all the tested samples (3.33 ± 1.00–6.81 ± 1.69 mg 100 g^−1^ DW), followed by zinc, while copper was recorded as the lowest concentration in all samples. The results showed that the Ajwa date was superior to Siwi and Sukkari in their content of macro- and micro-elements, with highly significant differences except for iron, where Sukkari and Siwi dates recorded a greater amount than the Ajwa dates. Siwi variety recorded higher iron content with 6.81 ± 1.69 mg/100 g^−1^ of DW.

The obtained results agreed with those described by Siddeeg et al. and Trabzuni et al. for the Sukkari date [25,54], by El-Sayed et al., El-Sohaimy and Hafez, and Ramadan et al. for the Siwi date [6,48,55], and by Assirey for the Ajwa date fruit [29]. On the other hand, our results were much higher than those observed by Al-Tamim, Assirey, and Selim et al. for Ajwa, Sukkari, and Siwi dates [29,49,50] and significantly higher when compared with those observed by Ali Alghamdi for different varieties of date fruits cultivated in Saudi Arabia [53]. The high concentrations of macro- and micro-elements present in the studied date varieties, which are necessary for growth and immunity, could be used to fortify children’s and nursing mothers’ meals and make them suitable for hypertension patients [56]. The observed variations among the studied dates could be due to the variety, soil type, irrigation, rain rate, amount of fertilizer, and ripening stage [52,57].

### 3.3. Amino Acid Composition of Date Fruits

Although date fruits are not a strong source of protein, date protein contains most of the essential amino acids needed for the human metabolic system, notably cell growth and regeneration [58]. The results of the amino acid analysis of the three studied varieties are presented in Table 3 and Supplementary Materials Figures S1–S3.

The non-polar amino acid proline was present in the highest amounts in the Siwi date and was the most prevalent amino acid in all studied date types. Additionally, the results revealed that all investigated dates contained high concentrations of aspartic acid, leucine, phenylalanine, and glutamine. Histidine recorded the lowest concentration among the identified amino acids in all date varieties. Ali et al., Hamad et al., and Khaled et al. obtained highly similar results for Ajwa and Sukkari dates grown in Saudi Arabia [23,59,60]. In another study by Assirey and El-Sayed et al., they indicated that glutamic acid is the predominant amino acid in Ajwa and Siwi dates [6,29].

It is observable that the Sukkari date had higher concentrations of aspartic acid and tyrosine than the other varieties, while it had the lowest concentration of leucine compared to the Siwi and Ajwa dates. Ajwa date was reported to have ample proteinogenic and non-proteinogenic amino acids [59]. The non-proteinogenic amino acids showed a remarkable effect on detoxifying harmful chemicals in the liver, leading to decreased creatinine in the human body. It is worth mentioning that the high concentrations of aspartic acid and tyrosine in a Sukkari variety play principal roles both as building blocks of proteins and as intermediates in metabolism. The consumption of this fruit may aid in reducing the consequences related to malnutrition [58].

### 3.4. Total Phenolics and Total Flavonoids Content (TPC and TFC)

It is well known that phenolic and flavonoid substances have a direct impact on biological processes in plants, including antioxidant [61], antibacterial, and antifungal activity [2]. As a result, the TPC and TFC in the four different extracts of the three date varieties were determined. As illustrated in Table 4, the TPC and TFC of all extracts varied significantly. The ethyl acetate extract (EtOAc) of Sukkari (29.61 ± 0.26 mg GAE·g^−1^ dried extract) had the highest TPC level, followed by Ajwa date EtOAc extract (23.97 ± 0.29 mg GAE g^−1^ dried extract) and Siwi date EtOAc extract (16.22 ± 0.30 mg GAE·g^−1^ dried extract), while the TPC of the EtOH/H_2_O 70%, MeOH/H_2_O 80%, and H_2_O in the Ajwa date were the highest (18.62 ± 0.29, 14.84 ± 0.29, and 11.65 ± 0.30, respectively) than the Sukkari and Siwi date in all extracts except the EtOAc extract.

The TFC ranged from 17.40 ± 0.26 mg RE·g^−1^ dried extract in EtOAc to 1.17 ± 0.26 mg RE·g^−1^ dried extract in H_2_O in the case of Sukkari, almost as much as the differences in phenolic contents. As displayed in Table 4, the greatest TFC of 17.40 ± 0.26 was shown in the EtOAc of Sukkari, followed by Ajwa date EtOAc extract (13.48 ± 0.29 mg RE·g^−1^ dried extract) and Siwi date EtOAc extract (13.06 ± 0.30 mg RE·g^−1^ dried extract), whereas the order of TFC in the distinct other extracts of the three varieties of dates (Ajwa, Siwi, and Sukkari) followed the sequence EtOH/H_2_O 70% extracts > MeOH/H_2_O 80% extracts > H_2_O extracts, with the TFC ranging from 10.28 ± 0.29 to 1.17 ± 0.26.

According to Saleh et al. [62], the H_2_O extract has much greater TPCs than the alcoholic extract, particularly in Ajwa dates, contrary to our result. In comparison to the studies of Al-Farsi et al., Mohamed et al., and Salah et al., the amounts of phenolics and flavonoids were greater in this study [62,63,64]. This variation could be attributed to genetic factors, ecological conditions, agronomic treatments, and the maturation phase; dates have higher phenolic contents, especially in earlier ripening stages [65]. Furthermore, the extraction method is critical for obtaining a sufficient quantity and quality of phenolic and flavonoid compounds, directly impacting their activity [66]. Therefore, much attention to the ultrasound-assisted extraction method (UAE) used in this work has recently been received for obtaining the highest yield of phenolic extracts from plant materials [16].

### 3.5. Antioxidant Potential of Date Extracts

The antioxidant effect of the three date varieties was measured using DPPH·. A significant variance among the date varieties was recorded regarding the type of solvent applied for extraction and the amounts and quality of phenolic and flavonoid components in each extract. Table 5 shows the free radical scavenging (FRS) ability of examined date fruits using the DPPH· assay.

Our results showed that the ethyl acetate extract of Sukkari fruits has the highest antioxidant activity with an IC_50_ value of 132.4 ± 0.3 μg mL^−1^, while the aqueous extract of Ajwa date fruits showed the lowest antioxidant effect with an IC_50_ value of 867.1 ± 0.3 μg mL^−1^. The EtOAc extract of all date varieties has the highest antioxidant activity of all the extracts of the three date varieties, as shown in Table 5. However, the H_2_O extract exhibited the lowest level of antioxidant activity among all the extracts of the three date varieties (Ajwa, Siwi, and Sukkari) (Table 5). Although it is noteworthy that the EtOAc extract possesses the largest amount of phenolic and flavonoid contents, Al-Farsi et al. revealed that the antioxidant properties of date fruits are directly related to the presence of high concentrations of phenolic compounds [64]. Furthermore, the analysis of the correlation between scavenging activity and phenolic and flavonoid contents exposed a strong adverse relationship between phenolic contents and DPPH· (r = −0.78), and flavonoid contents and DPPH· (r = −0.65). These strong adverse relationships advocate that the phenolic and flavonoid constituents are the main providers of the scavenging capability detected in tested date fruits. 

### 3.6. HPLC-DAD Analysis of Phenolic and Flavonoid Compounds in Ethyl Acetate Extracts

Based on the above analysis of the antioxidant effect, the TPC and TFC of the examined extracts of different date types and the EtOAc extract of the examined date fruits were analyzed using HPLC-DAD analysis, revealing the presence of different phenolic and flavonoid compounds that were identified through the comparison with the available standards according to UV data and the retention times (Table 6 and Supplementary Materials Figures S4–S6).

The major phenolic constituents of Ajwa date included p-hydroxybenzoic acid (59.127 µg·g^−1^), syringic acid (27.815 µg·g^−1^), and sinapic acid (22.860 µg·g^−1^). At the same time, the major phenolic compounds detected in Sukkari extract were p-hydroxybenzoic acid (81.34 µg·g^−1^), catechin (63.30 µg·g^−1^), chlorogenic acid (50.35 µg·g^−1^), ferulic acid (122.8 µg·g^−1^), sinapic acid (51.73 µg·g^−1^), and kaempferol (20.47 µg·g^−1^). Therefore, considering that major compounds have a concentration greater than 20 µg·g^−1^, we found that the Sukkari cultivar had six major compounds whereas the Ajwa cultivar had three, and according to this consideration, there were no compounds with a higher concentration in the Siwi cultivar. However, since there is a scarcity of research on phenolic and flavonoid constituents in ethyl acetate extract from date fruits, we could not compare it with earlier work.

The phenolic (phenolic acids and flavonoids) compounds in the ethyl acetate extract of Sukkari dates are present in concentrations significantly higher than their counterparts in Ajwa and Siwi dates. In addition, there are also detected compounds in the Sukkari date with high concentrations devoid of Ajwa and Siwi cultivar extracts. The following are the phenolic and flavonoid compounds detected in Sukkari extract alone, without the other extracts: (a) Kaempferol, which is effective in scavenging radicals and combating cancer [61,67], (b) Catechins, in which scientific studies have shown that their polyhydroxylated structure and the presence of specific structural features play a significant role in catechin’s antioxidant efficiency; and there are promising results of green tea catechin in cancer treatment, including breast, lung, stomach, and pancreatic cancers, as reported by several studies [68,69,70]. (c) Chlorogenic acid was reported as a potent antioxidant, anti-inflammatory, anti-obesity, anti-carcinogenic, and antibacterial [71,72,73,74]. (d) Ferulic acid is a powerful antioxidant with several biological activities, including anticancer properties against lung, breast, colon, and skin cancer, anti-inflammatory, and antimicrobial [75,76,77,78]. Based on the aforementioned, we can explain the outstanding effectiveness of Sukkari ethyl acetate extract as an antioxidant and anticancer agent compared to the other examined extracts.

### 3.7. Anticancer Activity of Various Extracts of Date Varieties

The cytotoxic activity of EtOAc, EtOH/H_2_O 70%, and MeOH/H_2_O 80% extracts at concentrations ranging from 62.5 to 500 μg·mL^−1^ after exposure to the tested date varieties for 48 h. was assessed in human cancer and normal cells using the sulphorhodamine-B (SRB) assay. Human osteosarcoma cell line (MG-63), human colon cancer cell line (HCT116), human breast cancer cell lines (MCF7 and MDA-MB-231), human hepatocellular cancer cell lines (HEPG2 and HuH7), human lung cancer cell lines (A549 and H460), and human normal melanocyte cell line (HFB4) were used for the screening.

**Figure 2:**
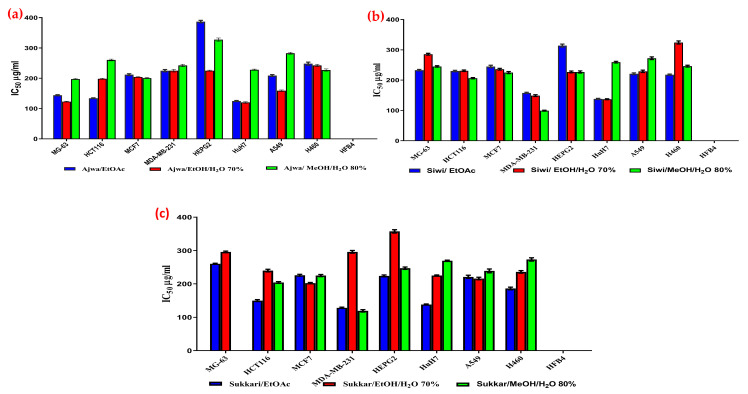
Cytotoxicity (IC_50_; µg/mL) of Ajwa extracts (**a**), Siwi extracts (**b**), and Sukkari extracts (**c**) against cancer cell lines.

Cytotoxicity of (IC_50_; µg·mL^−1^) all examined extracts against the tested cancer cell lines is shown in Figure 2a–c and Supplementary Materials Tables S1–S3. The used extracts exhibited potent to moderate anticancer activities against the aforementioned cancer cell lines in a source-dependent manner. The MeOH/H_2_O 80% extract of Siwi fruits exhibited the most potent anticancer activity (IC_50_= 99 ± 1.6 µg·mL^−1^), followed by the same extract of Sukkari fruits with an IC_50_ value of 119 ± 3.5 µg·mL^−1^ against the cell line of human breast cancer (MDA-MB-231). As a result, Siwi dates have a greater impact than expected. This effect on this type of cancer cell in particular may be due to components other than phenolic compounds, i.e., mono and sesqui-terpenes that are not detected herein in the Siwi date, that might be responsible for this effect [6].

To the best of our knowledge, our study is the second report that has dealt with the Siwi variety biologically and phytochemically. The Siwi variety was first examined by El-Sayed et al. [6], who reported the phytochemical profile and biological activity of four different extracts (MeOH/H_2_O 70%, EtOAc, n-butanol, and chloroform) in the Siwi date variety. They identified 20 monoterpenes and 5 sesquiterpenes, beside quercetin, and 1 phenolic acid, namely coumaric acid, in the non-polar solvents by GC-MS/ESIMS, which showed positive radical scavenging activity and moderate antibacterial activities against Gram (+) and Gram (−) bacteria. The four extracts displayed varying cytotoxic activities against HeLa and HEK293T cancer cell lines, with higher ethyl acetate and MeOH/H_2_O 70% extracts and slightly less for both n-butanol and chloroform extracts. These results are consistent with our findings and interpretations.

Several studies proposed that samples with IC_50_ values less than 125 μg·mL^−1^ could be a promising candidate for further advancement as a cancer therapeutic agent, whereas samples with IC_50_ values between 125 and 5000 μg·mL^−1^ were regarded to have moderate potential for future development into a cancer therapeutic agent [79,80,81,82,83]. Accordingly, all the examined extracts exhibited moderate to strong cytotoxic activity toward the examined cancer cell lines except the methanolic extract of the variety Sukkari, which has proven inactive against the human osteosarcoma cell line (MG-63). Due to the absence of cytotoxicity in the human normal melanocyte cell line (HFB4), these extracts exhibit a wide therapeutic window for cancer treatment.

Natural substances have a long history of being used as cancer prevention agents. It has chemo-preventive properties. Several studies have shown that numerous plant-based substances, including taxol, vincristine, and vinblastine, have potent anticancer activities with little or no adverse effects [20]. Ajwa dates are another naturally occurring fruit containing active substances with a wide therapeutic window for several disorders, such as anti-inflammatory, antioxidant, hepatoprotective, nephroprotective, and anticancer capabilities [32].

*Phoenix dactylifera* L. includes phenolic acids, carotene, polyphenols, anthocyanins, and tannins, among other phytochemicals. Another investigation on the phenolic content of Ajwa dates reported the existence of three significant phenols in pharmacology: catechin, caffeic acid, and rutin [84,85,86,87]. Therefore, the anticancer activities reported might be attributed to the incidence of flavonoids and phenolics in dates, which are high in phenolic compounds [14].

El et al. [82] reported that the cytotoxic impact of roasted date pits might be ascribed to the phenolic constituent contents and antioxidant properties. Research indicates that flavonoids may have a role in suppressing tumor cell growth and acting as chemopreventive agents against carcinogenesis in humans. Añón et al. [88] reported the protective effects of a range of phenolic acids and flavonoids on the cytotoxicity of CCl_4_ in rat hepatocytes.

Paranthaman [89] revealed that roasted date pits had a higher overall content of caffeine, flavonoids, and antioxidant capacity than raw date pits, which provide a pharmacologically active material depending on the dose, in agreement with our results. Therefore, in this investigation, extracts of three date varieties were judged to be a high-prospective source and a moderate-prospective source for several cancer cell lines.

### 3.8. Interrelationships among Studied Traits and Treatments

**Figure 3:**
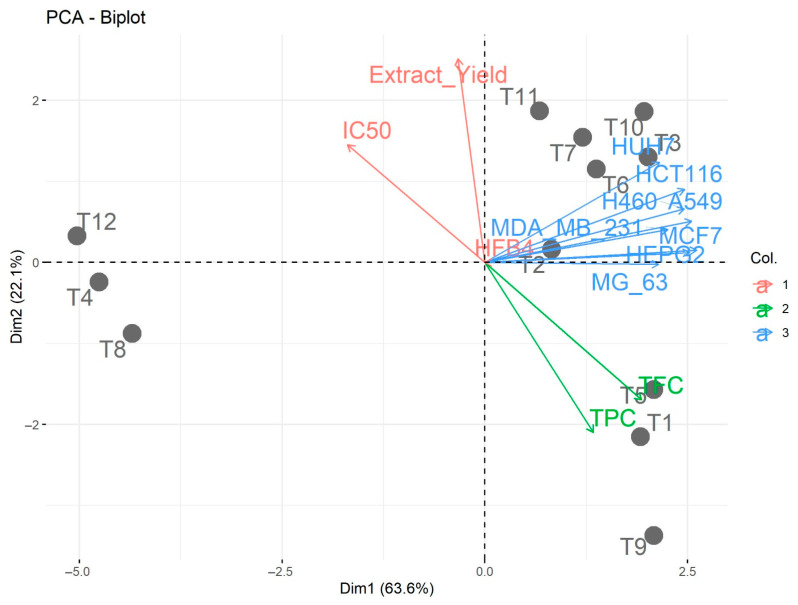
PCA biplot for the evaluated traits of Ajwa, Siwi, and Sukkari date fruit under four different extracts. T1: Ajwa × EtOAc; T2: Ajwa × EtOH/H_2_O 70%; T3: Ajwa × MeOH/H_2_O 80%; T4: Ajwa × H_2_O; T5: Siwi × EtOAc; T6: Siwi × EtOH/H_2_O 70%; T7: Siwi × MeOH/H_2_O 80%; T8: Siwi × H_2_O; T9: Sukkari × EtOAc; T10: Sukkari × EtOH/H_2_O 70%; T11: Sukkari × MeOH/H_2_O 80%; T12: Sukkari × H_2_O. TPC: total phenolic content; TFC: total flavonoid content.

Principal component analysis (PCA) was employed to evaluate the association among the investigated traits and treatments, as shown in Figure 3.

The first two PCAs (Dim 1 and Dim 2) accounted for 85.70% of the variability. Dim 1 explained 63.6% of the variation and was related to decreasing the polarity gradient from polar solvent (H_2_O extract) in all varieties to hydroalcoholic and non-polar solvent (EtOAc extract) (Figure 3). The increase from hydroethanolic to hydromethanolic had a small effect, as depicted by the small distance from cancer cell line treatments along Dim 1, while the distance in the multidimensional spaces of (T1 (Ajwa × EtOAc), T5 (Siwi × EtOAc) and T9 (Sukkari × EtOAc)) and (T4 (Ajwa × H_2_O), T8 (Siwi × H_2_O), and T12 (Sukkari × H_2_O)) were much more spread out, implying dissimilarity. Dim 1 divided the treatment levels into two groups: the cancer cell lines and TPC and TFC were situated on the positive side, but those of moderate and lower values were located on the negative side (Figure 3). Dim 2 explained 22.1% of the variation and seems to correspond with aqueous extracts, from the bottom (Ajwa T4 and Siwi T8) to the top (Sukkari T12). Aqueous extracts of three varieties of date, i.e., T4 (Ajwa), T8 (Siwi), and T12 (Sukkari) treatments were more dissimilar under cancer cell lines, TPC and TFC compared to T2 (Ajwa × EtOH/H_2_O 70%), T3 (Ajwa × MeOH/H_2_O 80%), T6 (Siwi × EtOH/H_2_O 70%), T7 (Siwi × MeOH/H_2_O 80%), T10 (Sukkari × EtOH/H_2_O 70%), and T11 (Sukkari × MeOH/H_2_O 80%) or TPC and TFC (T1 (Ajwa × EtOAc), T5 (Siwi × EtOAc) and T9 (Sukkari × EtOAc)). Cancer cell lines and their attributes were associated with TPC and TFC in the EtOAc extracts of three varieties of date tested (T1 (Ajwa), T5 (Siwi), and T9 (Sukkari)) in Dim 1, whereas extraction yields and IC_50_ were associated with polar extraction. The adjacent vectors of traits reflect a strong positive association, while vectors with larger angles prove a weak association, and opposite vectors (at 180 °C) reveal a negative relationship. A strong positive association was observed among various solvents (alcoholic and EtOAc) and all their attributes, while there was a negative association with extraction yields and IC_50_ values. As expected from the findings previously discussed, different solvents and related traits were positively correlated and opposite those of extraction yields and IC_50_ values. Moreover, a significant difference was detected between ethyl acetate extracts and other extracts tested. Hence, the PCA biplot supported the aforementioned presented findings.

## 4. Conclusions

The work aimed at studying the nutritional value, antioxidant, and anticancer activity effects of ethyl acetate, hydroethanol, hydromethanol, and aqueous fruit extracts of three date palm varieties (Ajwa, Siwi, and Sukkari), as well as the phytochemical profiles of their ethyl acetate extract only. The ethyl acetate extract of Sukkari fruits displayed the maximum antioxidant activity. Furthermore, all the examined extracts exhibited strong to moderate anticancer activities against the examined cancer cell lines in a source-dependent manner. Moreover, the PCA biplot reflects a strong positive association among various solvents (alcoholic and EtOAc) and all their attributes, while there was a negative association with extraction yields and IC_50_ values. This outcome hints that the biologically active components of the date fruits Ajwa, Siwi, and Sukkari may be effective natural anticancer medicines. Unlike current anticancer drugs, these findings open up the possibilities of using date palm fruit-derived extracts as a new anticancer strategy from safe and edible natural resources.

## Figures and Tables

**Table 1:** Chemical composition of date samples on a dry weight basis.

Component (%)	Ajwa	Siwi	Sukkari
Moisture	17.31 ±0.46 ^b^	19.18 ± 0.50 ^a^	18.13 ± 0.38 ^ab^
Total sugars	75.24 ± 1.75 ^b^	80.25 ± 2.02 ^a^	79.42 ± 2.90 ^a^
Non-reducing sugars	3.88 ± 0.02 ^b^	04.89 ± 0.01 ^a^	3.780 ± 0.00 ^b^
reducing sugars	71.55 ± 1.25 ^b^	75.36 ± 2.01 ^a^	75.64 ± 1.05 ^a^
Ash	3.86 ± 0.01 ^a^	2.14 ± 0.05 ^b^	2.480 ± 0.00 ^b^
Crud protein	3.25 ± 0.03 ^a^	1.970 ± 0.01 ^b^	2.260 ± 0.01 ^b^
Crud fibers	3.51 ± 0.04 ^a^	2.580 ± 0.00 ^b^	2.130 ± 0.02 ^b^
Crud lipids	0.97 ± 0.000 ^a^	1.230 ± 0.05 ^a^	1.050 ± 0.04 ^a^

Values are expressed as mean ± SD, *n* = 3. Means in the same row followed by the same letter are not significantly different at *p* < 0.05 using DMRT, while those with different letters are significantly different in the variety of Ajwa, Siwi, and Sukkari.

**Table 2:** Mineral content of dates varieties (Ajwa, Siwi, and Sukkari) investigated by ICP-OES (mg/100 g DW).

Variety	Ca	Na	Mg	K	Zn	Fe	Cu	P
Ajwa	128.79 ± 3.22 ^a^	45.35 ± 1.24 ^a^	151.17 ± 2.32 ^a^	1118.64 ± 4.27 ^a^	1.33 ± 0.01 ^a^	3.33 ± 1.00 ^b^	0.695 ± 0.51 ^a^	231.30 ± 6.25 ^a^
Siwi	55.73 ± 1.87 ^c^	37.15 ± 2.64 ^ab^	92.88 ± 1.11 ^b^	1040.26 ± 3.86 ^b^	0.93 ± 0.00 ^b^	6.81 ± 1.69 ^a^	0.612 ± 0.22 ^a^	167.18 ± 2.55 ^b^
Sukkari	64.12 ± 2.02 ^b^	30.54 ± 1.01 ^b^	91.597 ± 3.52 ^b^	1038.11 ± 3.45 ^b^	1.16 ± 0.02 ^ab^	6.11 ± 2.04 ^a^	0.37 ± 0.00 ^b^	109.92 ± 2.07 ^c^

Values are shown as mean ± SD, *n* = 3. Means in the same column followed by the same letter are not considerably different at *p* < 0.05 using DMRT, while those with different letters are statistically different in the variety of Ajwa, Siwi, and Sukkari.

**Table 3:** Amino acids contents of Ajwa, Siwi, and Sukkari date fruit (μg/g dry fruits).

Amino Acid	Ajwa	Siwi	Sukkari
Aspartic acid	105.521 ± 0.011	65.530 ± 0.31	122.018 ± 0.06
Glutamic acid	9.804 ± 0.0301	11.861 ± 0.00	8.250 ± 0.058
Serine	3.181 ± 0.0810	4.118 ± 0.036	3.059 ± 0.074
Histidine	0.9580 ± 0.0216	1.065 ± 0.005	1.568 ± 0.0289
Glycine	5.8510 ± 0.172	5.922 ± 0.289	5.407 ± 0.102
Threonine	2.6920 ± 0.034	2.080 ± 0.025	2.340 ± 0.041
Arginine	4.1491 ± 0.023	4.463 ± 0.082	6.909 ± 0.075
Alanine	5.501 ± 0.0000	5.506 ± 0.001	7.471 ± 0.007
Tyrosine	2.816 ± 0.0271	3.016 ± 0.013	10.805 ± 0.063
Cystine	n.d.	n.d.	n.d.
Valine	6.661 ± 0.0121	6.360 ± 0.021	9.366 ± 0.061
Methionine	8.222 ± 0.0502	8.334 ± 0.032	9.619 ± 0.016
Phenylalanine	17.212 ± 0.0070	15.868 ± 0.005	18.891 ± 0.091
Isoleucine	3.037 ± 0.0453	3.319 ± 0.055	5.931 ± 0.062
Leucine	42.145 ± 0.0130	41.639 ± 0.034	4.516 ± 0.057
Lysine	n.d.	n.d.	n.d.
Proline	673.744 ± 0.028	796.344 ± 0.018	681.476 ± 0.075

Values mean ± SD, *n* = 3. n.d.: not detected.

**Table 4:** Extraction yield, total phenolics, and total flavonoid contents of EtOAc, alcoholic, and aqueous extracts of date varieties.

Variety	Extract	Extract Yield (%)	TPC (mg GAE·g^−1^ Extract)	TFC (mg RE·g^−1^ Extract)
Ajwa	EtOAc	0.901 ± 0.17 ^j^	23.97 ± 0.29 ^b^	13.48 ± 0.29 ^b^
	EtOH/H_2_O 70%	44.36 ± 0.34 ^d^	18.62 ± 0.29 ^c^	10.28 ± 0.29 ^c^
	MeOH/H_2_O 80%	46.58 ± 0.25 ^c^	14.84 ± 0.29 ^e^	6.66 ± 0.29 ^e^
	H_2_O	32.10 ± 0.41 ^g^	11.65 ± 0.3 g^h^	5.42 ± 0.30 ^f^
Siwi	EtOAc	0.640 ± 0.11 ^k^	16.22 ± 0.30 ^d^	13.06 ± 0.30 ^b^
	EtOH/H_2_O 70%	42.36 ± 0.23 ^e^	12.35 ± 0.02 ^fg^	9.84 ± 0.021 ^c^
	MeOH/H_2_O 80%	45.70 ± 0.42 ^c^	12.70 ± 0.26 ^f^	6.42 ± 0.26 ^e^
	H_2_O	30.22 ± 0.36 ^h^	11.54 ± 0.3 ^gh^	3.97 ± 0.30 ^g^
Sukkari	EtOAc	1.101 ± 0.01 ^i^	29.61 ± 0.26 ^a^	17.40 ± 0.26 ^a^
	EtOH/H_2_O 70%	49.86 ± 0.28 ^b^	11.42 ± 0.28 ^h^	8.70 ± 0.28 ^d^
	MeOH/H_2_O 80%	51.20 ± 0.34 ^a^	10.80 ± 0.26 ^h^	5.77 ± 0.26 ^f^
	H_2_O	34.60 ± 0.29 ^f^	9.22 ± 0.26 ^i^	1.17 ± 0.26 ^h^

Values expressed as mean of triplicates ± SD (*p* < 0.05). Significant interactions between each variety and extracts are indicated with different lowercase letters (*p* ≤ 0.05) using DMRT.

**Table 5:** IC_50_ values of alcoholic and aqueous extracts of date varieties.

Variety	Extract	IC_50_ (μg·mL^−1^)
Ajwa	EtOAc	316.97 ± 0.29 ^c^
	EtOH/H_2_O 70%	470.06 ± 0.29 ^g^
	MeOH/H_2_O 80%	352.27 ± 0.29 ^d^
	H_2_O	867.07 ± 0.30 ^l^
Siwi	EtOAc	161.55 ± 0.30 ^b^
	EtOH/H_2_O 70%	631.69 ± 0.64 ^j^
	MeOH/H_2_O 80%	504.53 ± 0.26 ^h^
	H_2_O	396.37 ± 0.30 ^e^
Sukkari	EtOAc	132.40 ± 0.26 ^a^
	EtOH/H_2_O 70%	626.89 ± 0.28 ^i^
	MeOH/H_2_O 80%	425.62 ± 0.26 ^f^
	H_2_O	807.89 ± 0.26 ^k^

Values expressed as mean of triplicates ± SD (*p* < 0.05). Significant interactions between each variety and extracts are indicated with different lowercase letters (*p* ≤ 0.05) using DMRT.

**Table 6:** Chemical composition of the EtOAc extracts of date varieties.

No.	Rt (min)	Compound	Quantification (µg·g^−1^)		
			Ajwa	Siwi	Sukkari
1	4.0	Gallic acid	5.30 ± 0.00	n.d.	8.30 ± 0.01
2	6.7	Protocatechuic acid	n.d.	n.d.	16.68 ± 0.00
3	10.0	*p*-hydroxybenzoic acid	59.13 ± 0.02	n.d.	81.34 ± 0.01
4	12.0	Catechin	n.d.	n.d.	63.31 ± 0.00
5	12.3	Chlorogenic acid	5.26 ± 0.00	n.d.	50.35 ± 0.01
6	13.5	Caffeic acid	3.04 ± 0.03	n.d.	13.01 ± 0.02
7	14.4	Syringic acid	27.82 ± 0.01	n.d.	17.11 ± 0.00
8	16.2	Vanillic acid	n.d.	n.d.	7.07 ± 0.00
9	20.5	Ferulic acid	5.41 ± 0.01	n.d.	122.83 ± 0.01
10	21.2	Sinapic acid	22.86 ± 0.00	2.97 ± 0.01	51.74 ± 0.00
11	26.2	*p*-coumaric acid	n.d.	14.81 ± 0.00	n.d.
12	35.8	Cinnamic acid	2.96 ± 0.02	n.d.	7.82 ± 0.01
13	36.3	Quercetin	5.88 ± 0.00	n.d.	7.35 ± 0.00
14	40.5	Kaempferol	n.d.	n.d.	20.48 ± 0.00

Values expressed as mean of triplicates ± SD (*p* < 0.05). n.d.: not detected.

## Data Availability

The data used to support the findings of this study are included within the article.

## Supplementary Materials

The following supporting information can be downloaded at: https://www.mdpi.com/article/10.3390/metabo13070816/s1. Figures S1–S3: Separated amino acids from Ajwa, Siwi, and Sukkari date fruits HPLC chromatograms. Figures S4–S6: HPLC-DAD analysis Chromatogram of Ajwa, Siwi, and Sukkari ethyl acetate extracts. Tables S1–S3: Cytotoxicity (IC_50_; µg/mL) of Ajwa, Siwi, and Sukkari examined extracts on different cell lines.
