# Supplementary material for: Antioxidant and Anticancer Assessment and Phytochemical Investigation of Three Varieties of Date Fruits
Source: Metabolites. 2023 Jul 3;13(7):816. doi: 10.3390/metabo13070816 (PMC10386203; doi:10.3390/metabo13070816)
Supplement: Supplementary file 1 [file metabolites-13-00816-s001.zip › metabolites-2445458-supplementary.pdf]

Supplementary Materials

Table of Contents

Figure S1. HPLC Chromatogram of separated amino acids from Ajwa date fruit.
Figure S2. HPLC Chromatogram of separated amino acids from Siwi date fruit.
Figure S3. HPLC Chromatogram of separated amino acids from Sukkari date fruit.
Figure S4. HPLC-DAD analysis Chromatogram of Ajwa Ethyl acetate extract.
Figure S5. HPLC-DAD analysis Chromatogram of Siwi Ethyl acetate extract.
Figure S6. HPLC-DAD analysis Chromatogram of Sukkari Ethyl acetate extract.
Table S1. IC_{50} of Ajwa examined extracts on different cell lines.
Table S2. IC_{50} of Siwi examined extracts on different cell lines.
Table S3. IC_{50} of Sukkari examined extracts on different cell lines.

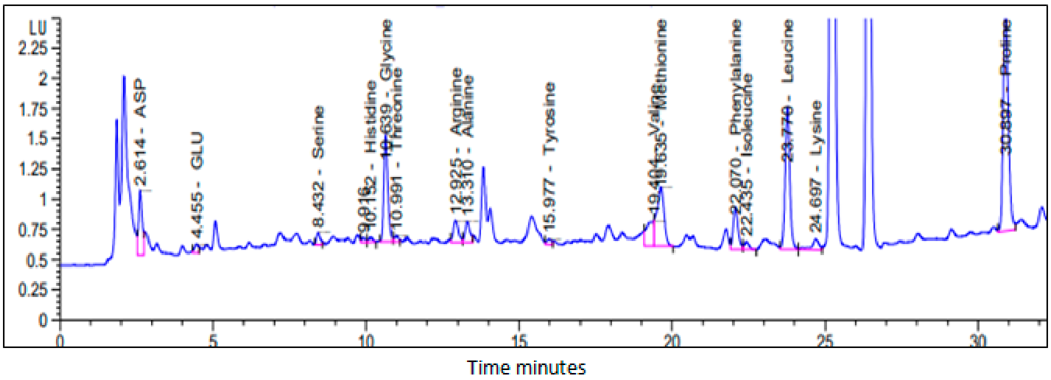

Figure S1. HPLC Chromatogram of separated amino acids from Ajwa date fruit.

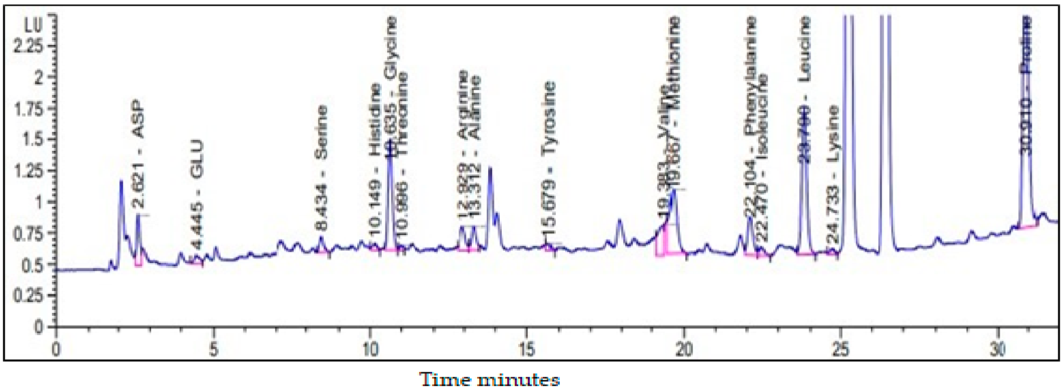

Figure S2. HPLC Chromatogram of separated amino acids from Siwi date fruit.

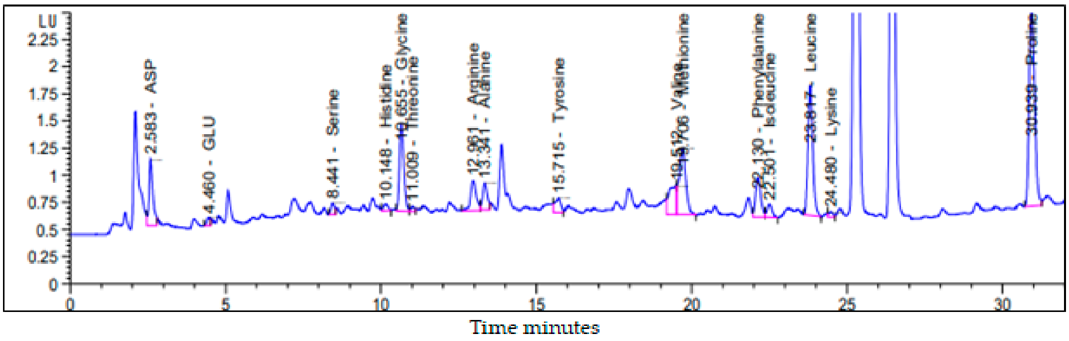

Figure S3. HPLC Chromatogram of separated amino acids from Sukkari date fruit.

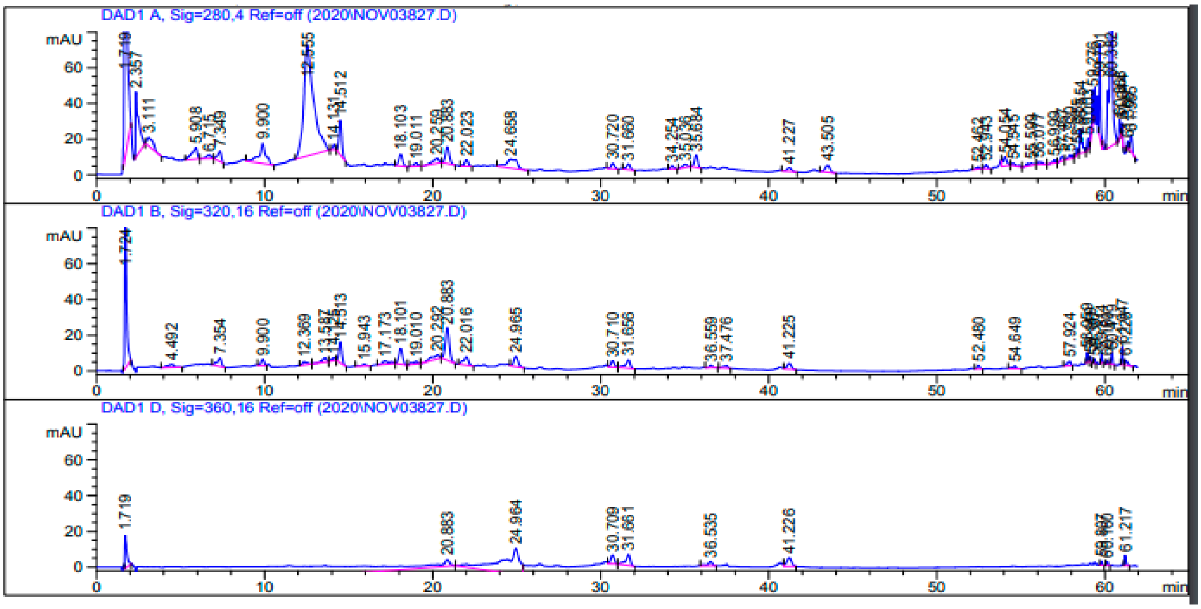

Figure S4. HPLC-DAD analysis Chromatogram of Ajwa Ethyl acetate extract.

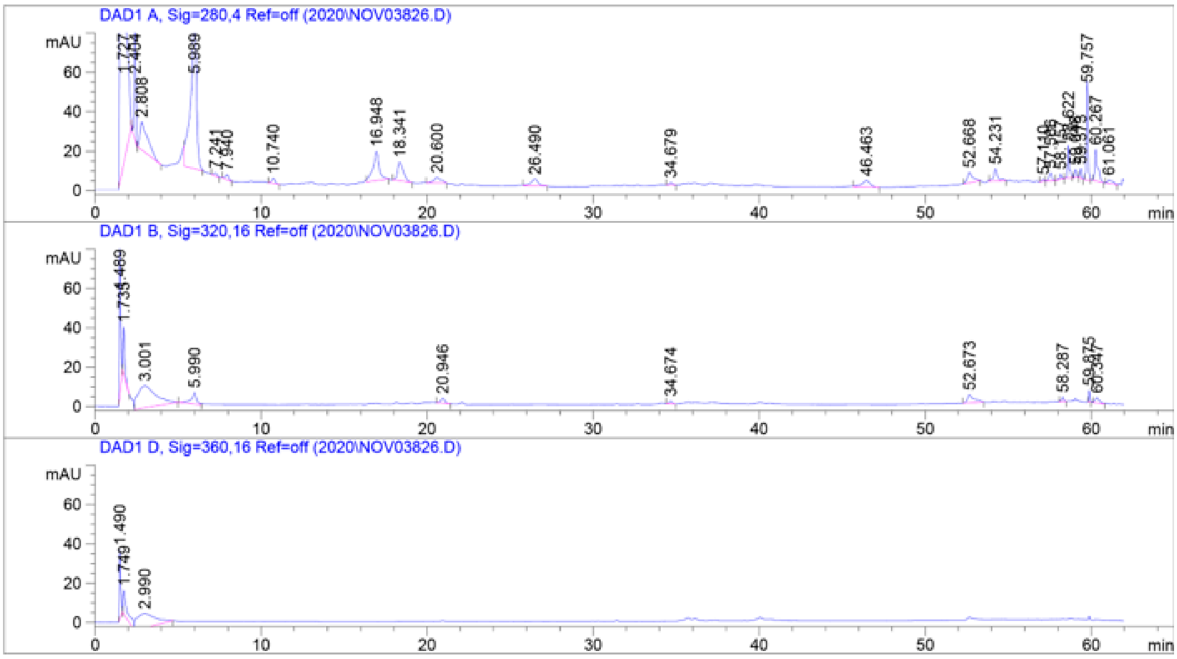

Figure S5. HPLC-DAD analysis Chromatogram of Siwi Ethyl acetate extract.

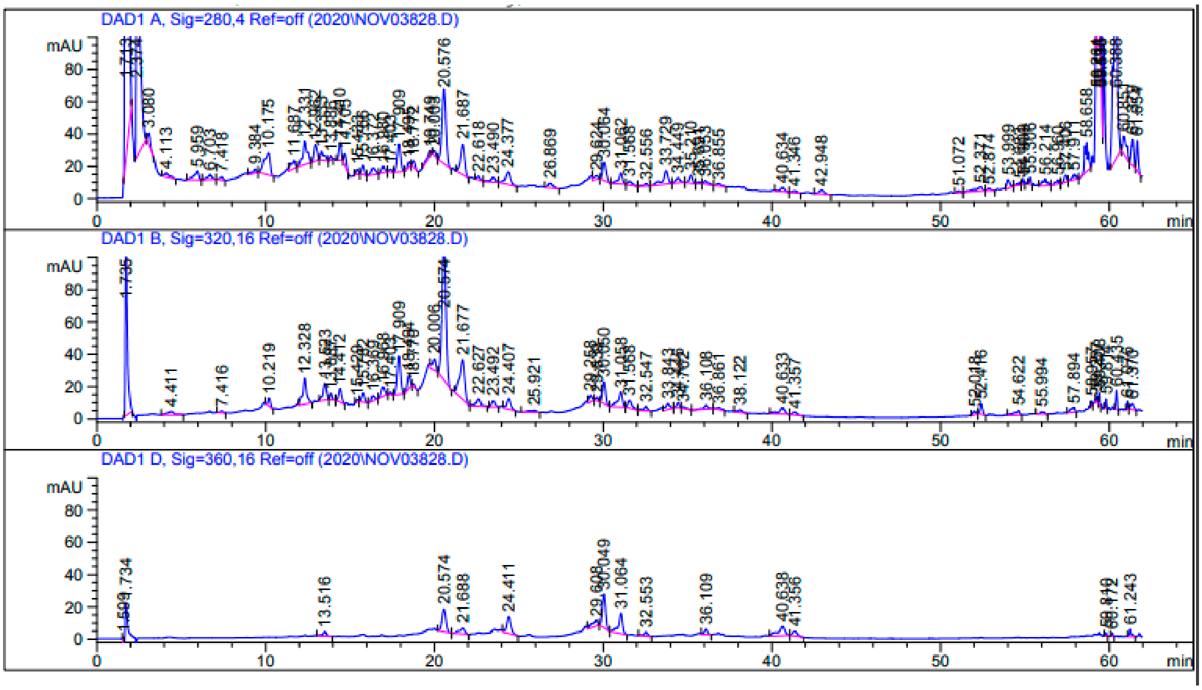

Figure S6. HPLC-DAD analysis Chromatogram of Sukkari Ethyl acetate extract.

Table S1. Cytotoxicity (IC_{50}; µg/ml) of Ajwa examined extracts on different cell lines.

Cancer cell line	Ajwa/ EtOH	Ajwa/ MeOH	Ajwa/ EtOAc
MG-63	123 ± 1.5	197 ± 2	144 ± 1.9
HCT116	198 ± 1.8	260 ± 3	134 ± 1.5
MCF7	204 ± 1.7	200 ± 2	212 ± 3
MDA-MB-231	225 ± 4.5	242 ± 4	225 ± 3.8
HEPG2	225 ± 2	327 ± 5.6	387 ± 4
HuH7	120 ± 3	228 ± 2.2	125 ±1.8
A549	159 ± 2.5	282 ± 3	209 ± 3
H460	242 ± 4	227 ± 4	248 ± 5
HFB4	-	-	-

Table S2. Cytotoxicity (IC_{50}; µg/ml) of Siwi examined extracts on different cell lines.

Cancer cell line	Siwi/ EtOH	Siwi / MeOH	Siwi/ EtOAc
MG-63	285 ± 3	245 ± 2.2	233 ± 3.4
HCT116	231 ± 2.5	206 ± 2	230 ± 2.4
MCF7	236 ± 2.9	225 ± 3.5	245 ± 4
MDA-MB-231	149 ± 3	99 ± 1.6	158 ± 2
HEPG2	227 ± 3	227 ± 3.6	314 ± 5
HuH7	137 ± 1.6	259 ± 2.8	138 ± 2
A549	229 ± 4	272 ± 4.5	221 ± 3
H460	324 ± 5	246 ± 3	218 ± 2
HFB4	-	-	-

Table S3. Cytotoxicity (IC_{50}; μg/ml) of Sukkari examined extracts on different cell lines.

Cancer cell line	Sukkari / EtOH	Sukkari / MeOH	Sukkari / EtOAc
MG-63	296 ± 2.5	-	260 ± 1.7
HCT116	240 ± 4	204 ± 2.6	150 ± 3
MCF7	202 ± 2	225 ± 3.3	226 ± 2.8
MDA-MB-231	296 ± 4.4	119 ± 3.5	128 ± 2
HEPG2	357 ± 5	247 ± 3.4	224 ± 3
HuH7	225 ± 2	269 ± 2	138 ± 1.9
A549	216 ± 4	239 ± 5.2	221 ± 5
H460	236 ± 4	273 ± 5	186 ± 4
HFB4	-	-	-
